# Synthesis, structure, and luminescent properties of a family of lanthanide-functionalized peroxoniobiophosphates

**DOI:** 10.1038/s41598-017-10811-2

**Published:** 2017-09-06

**Authors:** Haiying Wang, Jing Li, Junjun Sun, Yaya Wang, Zhijie Liang, Pengtao Ma, Dongdi Zhang, Jingping Wang, Jingyang Niu

**Affiliations:** Henan Key Laboratory of Polyoxometalate Chemistry, College of Chemistry and Chemical Engineering, Henan University, Kaifeng, Henan 475004 P. R. China

## Abstract

Eight new lanthanide derivatives containing 6-peroxoniobio-4-phosphate building block, [Ln^III^(H_2_O)_6_]_2_[H_4_(NbO_2_)_6_P_4_O_24_]·nH_2_O [Ln = Eu (**1**), Gd (**2**), Tb (**3**), Dy (**4**), Ho (**5**), Er (**6**), Tm (**7**), Yb (**8**), **1**–**5**, **7**, **8** n = 12; **6** n = 9], have been successfully obtained using an *in-situ* strategy and fully characterized in the solid state by single-crystal X-ray diffraction, IR spectra, TG-MS, PXRD. Structural analyses indicate that these isostructural polyanions **1**–**8** consist of one [P_4_(NbO_2_)_6_O_24_]^10−^ (**P**_**4**_ **(NbO**_**2**_ **)**_**6**_) clusters and two pendant Ln^3+^ cations. In these compounds, **P**_**4**_(**NbO**_**2**_ **)**_**6**_ clusters are connected by lanthanide cations to form extended two-dimensional architectures. The approach takes advantage of the ability of *in-situ* formed **P**_**4**_(**NbO**_**2**_ **)**_**6**_ cluster to build frameworks by using it as ligands to lanthanide ions. The photoluminescence (PL) and lifetime decay behaviors of **1**, **3** and **4** in solid state have been performed at room temperature. The PL emission of **1**, **3** and **4** is mainly derived from the characteristic ^5^D_0_→^7^F_J_ (J = 1, 2, 3, 4), ^5^D_4_→^7^F_J_ (J = 6, 5, 4, 3) and ^4^F_9/2_→^6^H _J_ (J = 15/2, 13/2, 11/2) transitions of the Eu^III^, Tb^III^ and Dy^III^ cations, respectively.

## Introduction

Polyoxometalates (POMs)^1, 2^ are a unique family of polynuclear anionic metal oxo clusters with properties suitable for many potential applications in catalysis, magnetism, biomedicine, materials science, and nanotechnology^3–5^. Whilst many Mo and W POM clusters can be built in a controlled way, the designed assembly of polyoxoniobate (PONb) is more difficult due to its inherent stability at higher pH. Till 2002, Nyman *et al*. discovered the first Keggin-type heteropolyoxoniobate anion [SiNb_12_O_40_]^16−^ ^6^, and thereafter, PONb species has become one of the most widely investigated families of molecules in the POM family^7–27^. However, the researches of PONb clusters are mainly focused on alkaline system owing to the basic nature of the hexaniobate Lindqvist ion [Nb_6_O_19_]^8−^ (**Nb**_**6**_)^7, 12^, the most common used starting material in PONb chemistry. In this context, the amphoteric or complexed transition-metal (TM) can be introduced to obtain novel heterometallic PONbs^12, 22–27^. Nevertheless, most of the reported TM-containing PONb derivatives are synthesized by hydrothermal or hydrothermal-diffusion methods. In contrast, some Nb-substituted polyoxotungstates of the classic Lindqvist or Keggin derivatives has been produced under acidic solution in the presence of H_2_O_2_^28–31^. Recently, we have successfully manipulated the reaction of **Nb**_**6**_ cluster under acidic condition by an *in-situ* synthetic strategy^19–22^. In this respect, a series of novel oligomeric derivatives have been successfully isolated, for example, {[P_2_W_12_Nb_6_(O_2_)_4_O_57_]_2_}, {[P_2_W_12_Nb_6_O_61_]_4_[Nb_4_O_10_]} and {P_2_W_12_Nb_7_O_63_}_4_[Nb_4_O_10_]}, all of these polyanions were obtained by using the *in-situ* formed cluster [P_2_W_12_(NbO_2_)_6_O_56_]^12−^ (P_2_W_12_(NbO_2_)_6_) as second building block (SBU)^19, 20^. In fact, the ability of these oxo-terminal O_t_(Nb) groups to react with TM was also recently demonstrated in the examples of {[P_2_W_12_Nb_6_]_6_Mn_15_}^21^ and {[SiW_9_Nb_3_]_3_Mn}^22^. This is because the substitution of W^VI^ by the lower Nb^V^ leads to an increased basicity of the oxygen atoms and thus has a strong nucleophilicity.

Despite these work, we are yet to introduce hetero atom into **Nb**_**6**_ acidic system to design and synthesize novel hetero-containing PONbs: in 2014 we reported a new As^V^-containing polyoxoniobate {As_2_Nb_4_(O_2_)_4_}^32^. Its structure resembles that of the peroxo-Nb_6_ cluster^33^, in which two peroxo-Nb groups are substituted by two As ligands. Very recently, we also obtained a 6-peroxoniobo-4-phosphate cluster isolated as guanidinium salt ((CN_3_H_6_)_6_[H_4_P_4_Nb_6_(O_2_)_6_O_24_]∙4H_2_O)^34^, which has the same polyanion TMA_3_[H_7_Nb_6_P_4_(O_2_)_6_O_24_]·7H_2_O reported previously by Casey *et al*. in 2015^15^. These results drive us to the development of a new *in-situ* formed SBU system, and suggested that it is possible to design and synthesize novel TM or lanthanide ion derivatives by using the *in-situ* formed **P**_**4**_(**NbO**_**2**_ **)**_**6**_ cluster as second building block.

On the other hand, it should be noted that even though the class of PONb-based lanthanide derivatives was pioneered by Yamase and Naruke as early as 1994^35^, only a few lanthanide-containing PONb clusters have been characterized so far (Table S1). Besides the above-mentioned isostructural polyoxometallolanthanoates, {[Ln_3_O(OH)_3_(OH_2_)_3_]_2_Al_2_(Nb_6_O_19_)_5_} (Ln = Eu, Tb, Er, Lu, Tb_4.3_Eu_1.7_)^35–38^, Liu *et al*. reported a series of lanthanide derivatives based on saturated W/Nb mixed-addenda POM in 2012, including {(P_2_W_15_Nb_3_)_4_Ln_6_} (Ln = Ce^3+^, Eu^3+^)^39^, {(GeW_9_Nb_3_)_n_Eu} (n = 2, 4), {(GeW_9_Nb_3_)_4_Cs(SO_4_)Eu_5_}, {(GeW_9_Nb_3_)_4_Cs_2_Eu_4_}, {(GeW_9_Nb_3_)_4_Eu_5.5_} and {GeW_9_Nb_3_Eu_1.25_}^40^. Very recently, Zheng *et al*. communicated the first series of giant Nb-W-Ln heterometallic POMs, {Ln_12_W_12_(Nb_6_O_19_)_12_} (Ln = Y, La, Sm, Eu, Yb)^14^.

Herein, we report the formation of a new family of phosphoniobate-based lanthanide derivatives, [Ln^III^(H_2_O)_6_]_2_[H_4_(NbO_2_)_6_P_4_O_24_]·nH_2_O [Ln = Eu (**1**), Gd (**2**), Tb (**3**), Dy (**4**), Ho (**5**), Er (**6**), Tm (**7**), Yb (**8**), **1**–**5**, **7**, **8** n = 12; **6** n = 9], making use of the *in-situ* formed **P**_**4**_(**NbO**_**2**_ **)**_**6**_ cluster as second building block. Their crystal structures have been solved by single-crystal X-ray diffraction and further characterized by IR spectra, powder X-ray diffraction (PXRD), and thermogravimetric-mass analyses (TG-MS). Structural analyses show that compounds **1**–**8** are isostructural and consist of a 6-peroxoniobio-4-phosphate [H_4_(NbO_2_)_6_P_4_O_24_]^6−^ fragment and two pendant [Ln(H_2_O)_6_]^3+^ cations, forming an interesting two-dimensional plane and representing the rare PONb-based lanthanide derivatives. In addition, the luminescent properties of **1**, **3** and **4** have been investigated systematically.

## Results and Discussion

Lindqvist type [Nb_6_O_19_]^8−^ (**Nb**_**6**_) anions are known to be stable above pH 10.5^7^, a state in which lanthanide ions are easy to hydrolysis and thus make it difficult to investigate polyoxoniobate-lanthanide (PONb-Ln) materials. In this paper, a new strategy for the synthesis of PONb-Ln derivatives has been developed. Its major experimental strong point is the facile preparation of target compounds from *in-situ* formed **P**_**4**_(**NbO**_**2**_ **)**_**6**_ building block from acidic aqueous solution. Eight new [Ln^III^(H_2_O)_6_]_2_[H_4_(NbO_2_)_6_P_4_O_24_]·nH_2_O compounds are all synthesized under the similar reaction conditions. The procedure involves forming two solutions. K_7_H[Nb_6_O_19_]·13H_2_O (0.55 g, 0.40 mmol) was dissolved in a solution consisting of 4.0 mL of 30% aqueous H_2_O_2_ and 36 mL of water, acidified to pH ca. 2.5 *via* addition of 1.0 mL H_3_PO_4_ (3.0 M), being as solution A. While solution B comprises LnCl_3_·nH_2_O (4.0 mmol) in 2.0 mL of water, which is added into solution A and the pH of the resultant mixture is adjusted to 1.2–2.0, depending on the lanthanide metal used. The mixtures are then heated at 90 °C for 4 hours and filtered and then left at room temperature to crystallize. Single crystals are collected after about two weeks with the average yield 10–20% based on niobium.

**Figure 1:**
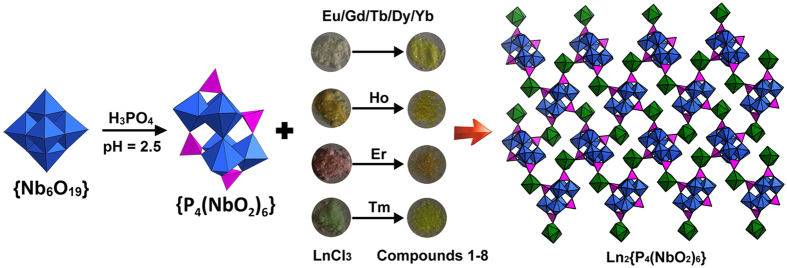
The synthetic route of **1**–**8**, highlighting the colour of LnCl_3_ and compounds.

Interestingly, the colour of the hexaniobate solution turns from colorless to bright-yellow with the addition of phosphoric acid, suggesting the formation of peroxo {NbO_2_} group. This is common in the previous {NbO_2_}-substituted polyoxotungstate chemistry^20, 31^, and also reinforced by the fact that the solution comprising **P**_**4**_(**NbO**_**2**_ **)**_**6**_ is intensely yellow, whereas the solution turns to colorless quite rapidly with the addition of NaHSO_3_. Importantly, the color of the resultant compounds **1**, **2**, **3**, **4** and **8** is almost same to that of **P**_**4**_(**NbO**_**2**_ **)**_**6**_ owing to the corresponding colorless lanthanide chloride, while the color of compounds **5**, **6** and **7** is somewhat different, depending on the color of lanthanide chloride used (Fig. 1).

There are several aspects of the synthetic conditions that can influence the formation of these clusters, taking compound **1** as example, a series of reaction sets under different experimental conditions of pH, temperature, heating time and the mole ratio of Eu^III^/Nb_6_ were investigated. If the solution of the reactants were left to heat at 90 °C for less than 2 h, we have been unable to isolate single crystals of compound **1**, however, it was found that there was no obvious increase in the yields when the heating time was more than 4 h. In addition, the reaction is not particularly sensitive to the amount of Eu^III^ because **1** could be obtained with the mole ratios of Eu^III^/Nb_6_ ranging from 6:1 to 16:1, with 10:1 giving the highest yield. It is worth mentioning here that compounds **1**−**8** can be isolated without the need for extra cations, different from an essential templating role of Cs^+^ in the formation of Keggin and Wells-Dawson-type niobium-substituted polyoxotungstates^28–31, 41–43^.

**Figure 2:**
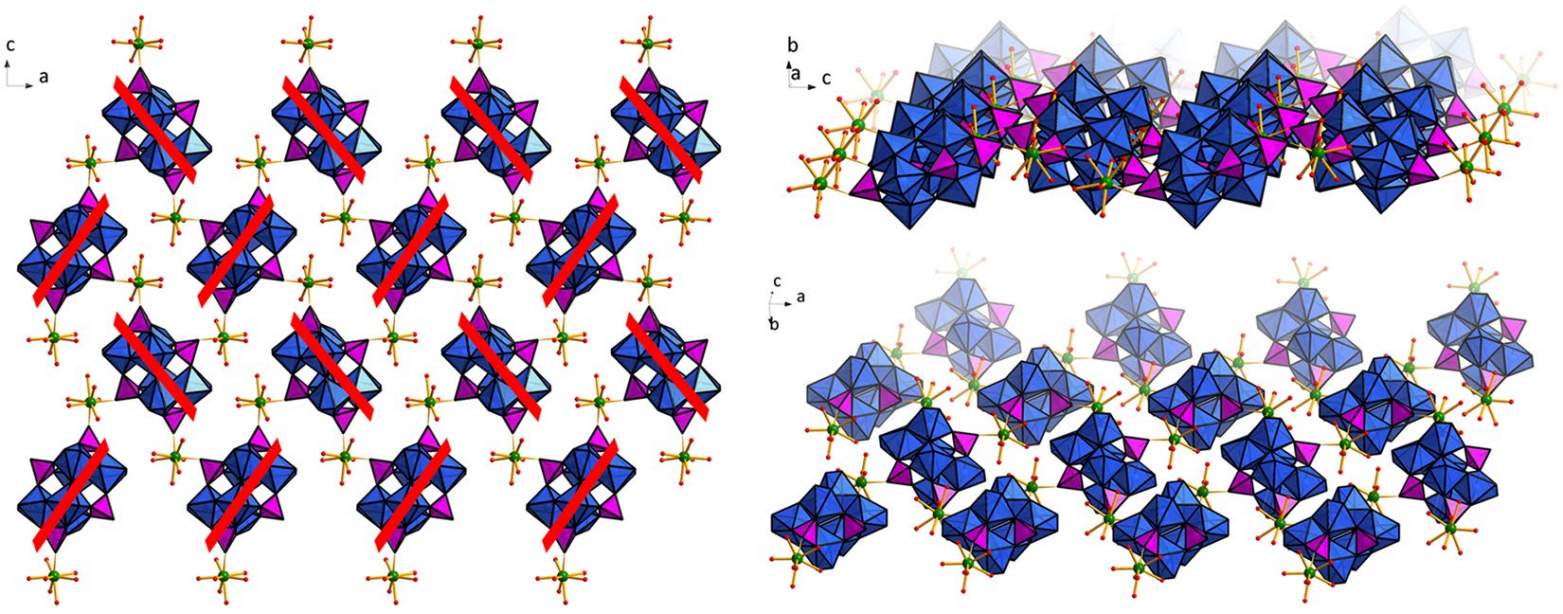
Representations of the 2D framework found in the structure of **1** from different directions. All solvent water molecules have been omitted for clarity. Color code: NbO_7_ blue polyhedral, PO_4_ pink polyhedral, EuO_8_ green dodecahedral, Nb blue spheres, P pink spheres, Eu green spheres.

Single-crystal X-ray diffraction analysis reveals that **1**−**8** are isostructural and comprise a neutral [Ln^III^(H_2_O)_6_]_2_[H_4_(NbO_2_)_6_P_4_O_24_] (Ln_2_P_4_(NbO_2_)_6_, Ln = Eu (**1**), Gd (**2**), Tb (**3**), Dy (**4**), Ho (**5**), Er (**6**), Tm (**7**), Yb (**8**)) subset and some lattice water molecules. The self-assembly of all presented crystal structures can be traced back to coordinative bonding forces and the lanthanide ions are coordinated to POMs, connecting them to two-dimensional frameworks (Fig. 2). All of the compounds crystallize in the space group orthorhombic *Pbca* and with almost identical unit cell dimensions. This is unsurprising given that the only difference between the clusters is the lanthanide metal present, and thus the structural description is only exemplified by **1**.

**Figure 3:**
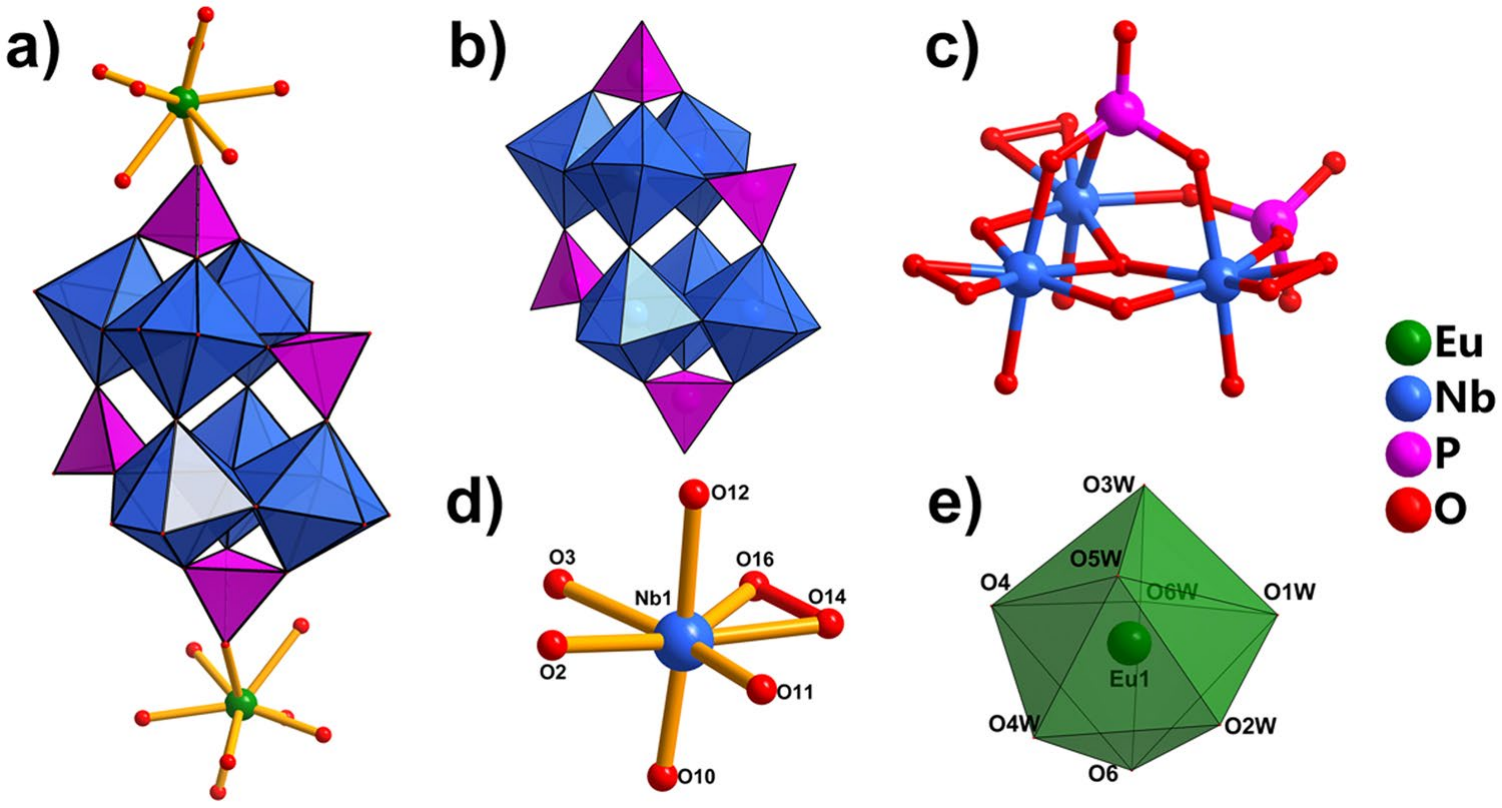
Ball-and-stick/polyhedral representations of Eu_2_P_4_(NbO_2_)_6_ (**a**), P_4_(NbO_2_)_6_ (**b**), P_2_Nb_3_ (**c**) and coordination environment of NbO_7_ (**d**) and EuO_8_ (**e**). All solvent water molecules have been omitted for clarity. Color code: NbO_7_ blue polyhedral, PO_4_ pink polyhedral, EuO_8_ green dodecahedral, Nb blue spheres, P pink spheres, Eu green spheres, O red spheres, peroxo bond red.

Structurally, the architecture Eu_2_P_4_(NbO_2_)_6_ consists of a 6-peroxoniobo-4-phosphate (**P**_**4**_ **(NbO**_**2**_ **)**_**6**_) cluster with two supporting [Eu(H_2_O)_6_]^3+^ fragments on both sides (Fig. 3a). Each **P**_**4**_ **(NbO**_**2**_ **)**_**6**_ cluster is linked to four [Eu(H_2_O)_6_]^3+^ fragments by four Eu–O–P bridges, whereas every [Eu(H_2_O)_6_]^3+^ moiety is bound to two **P**_**4**_ **(NbO**_**2**_ **)**_**6**_ clusters by two Eu–O–P bridges, resulting in a two-dimentional framework. Therefore, this subset should be formulated as Eu_2_P_4_(NbO_2_)_6_. To our knowledge, this is the first observation of the peroxoniobophosphate-based lanthanide derivatives in POM chemistry, although there are many examples of Mo, W POM-based lanthanide aggregates.

It is worth noting that the polyanion **P**_**4**_ **(NbO**_**2**_ **)**_**6**_ resembles structurally the previously reported [H_7_Nb_6_P_4_O_24_(O_2_)_6_]^3−^ (**1′**) cluster of Casey and co-authors^15^. This centrosymmetric cluster can be viewed as two **P**_**2**_ **Nb**_**3**_ units fused by two Nb‒*µ*_2_-O‒Nb and two P‒*µ*_2_-O‒Nb bridges (Fig. 3b). The **P**_**2**_ **Nb**_**3**_ can be regarded as a peroxohexaniobate^33^ with a contiguous longitudinal strip of three Nb(O_2_) groups (one on equatorial position and two on axial position) replaced by two PO_4_ groups (Figs 3c and S1). In **1**, each of the six Nb atoms is ligated by one *µ*_3_-O bridging atom, four *µ*_2_-O bridging atoms, and one terminal peroxo group, giving a pentagonal bipyramid geometry (Fig. 3d). As shown in Table S2, the average Nb–O and Nb–O_p_ bond length is in the range of 1.863(5)–2.110(5) and 1.912(9)–1.969(7) Å, respectively, and the average O_p_–Nb–O_p_ angle is 43.63°, not significantly different from those in **1′** reported previously^15^. The remaining sites in the coordination sphere of the lanthanide ions are occupied by six terminal aqua ligands (Eu–O: 2.275(6)–2.504(7) Å, Table S2), resulting in an eight-coordinated dodecahedral geometry (Fig. 3e). Additionally, all the P atoms exhibit conventional tetrahedral coordination polyhedra, and the P–O bond lengths are in the range of 1.496(6)–1.526(6) Å (Table S2), which is shorter than those in **1′**, where P–O lengths are 1.508(2)–1.561(2) Å^15^. The peroxo group bond lengths, O–O, range from 1.389(12) to 1.49(19) Å with the mean value 1.45 Å (Table S2), which is shorter than that for hydrogen peroxide published in 1951 (1.49 Å)^44^.

**Figure 4:**
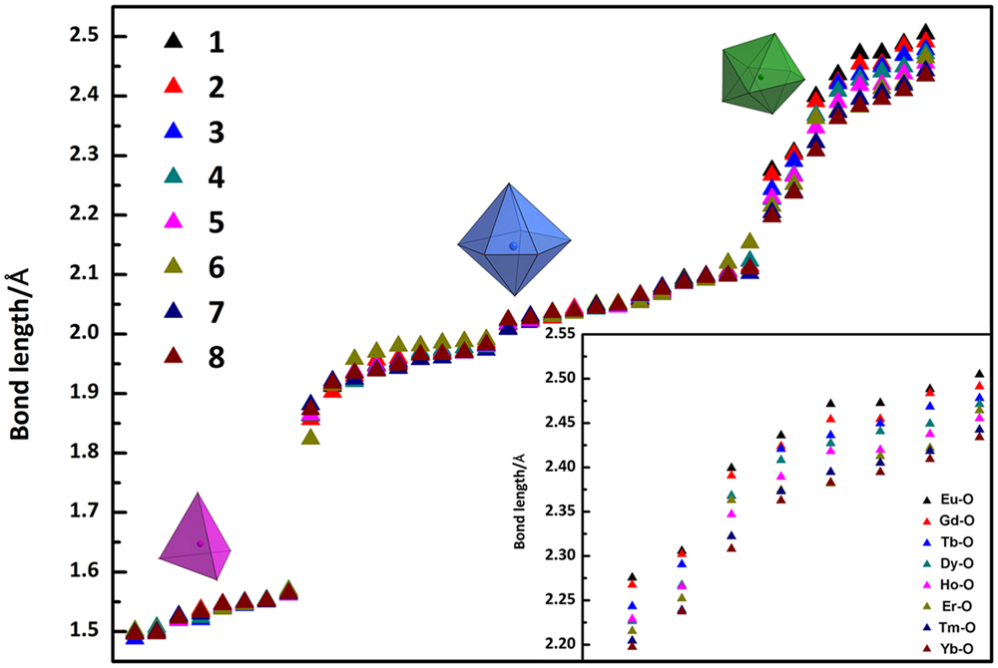
Comparison of the metal–oxygen bond lengths in **1**–**8**. Inset figure is Ln–O bond lengths of **1–8**.

The metal–oxygen bond lengths in **1**−**8** are sorted and plotted in the order of their lengths (Fig. 4), it can be clearly seen that the bond lengths of Nb-peroxo and Nb‒*µ*_3_‒O in **1**−**8** are almost the same. The Ln‒O bonds length are gradually reduced, which are generally in agreemnt with the ion radius trend in the lanthanide elements.

Bond valence sum (BVS) calculations^45^ are carried out on all the Ln, Nb, P and O centers (Table S3) and the results show that all the Ln, Nb and P atoms are in the +3, +5 and +5 oxidation states, respectively. The BVS values of the *µ*_2_-O oxygen atoms bridging Nb1–Nb2 (O3) are in the range of 1.29–1.31, suggesting that these oxygen atoms are monoprotonated. In addtition, charge-balance considerations with counter cations suggested that compounds **1**−**8** should contain the two additional protons, and we think these two protons are delocalized in the polyoxoanions on the basis of the previous studies by Nyman and Niu^46, 47^.

The Fourier transform infrared spectra (FT-IR) of **1**–**8**, (CN_3_H_6_)_6_[H_4_P_4_Nb_6_(O_2_)_6_O_24_]∙4H_2_O^34^ and K_7_H[Nb_6_O_19_]·13H_2_O (**Nb**_**6**_) were recorded in the range of 4000–450 cm^−1^ (Figures S2–3). As expected, the overall IR spectra of **1**–**8** are almost the same because of the isostructural nature (Table 1). All compounds **1**–**8** exhibit strong and medium bands in the range of 1200–1000 cm^−1^, associated with antisymmetric stretching of the P–O bond^15^. However, in comparison with the IR spectrum of the isolated (CN_3_H_6_)_6_[H_4_P_4_Nb_6_(O_2_)_6_O_24_]∙4H_2_O material, *ν*(P–O) and *ν*(Nb–O–Nb) vibration frequencies for **1**–**8** have different shifts, which may be due to the coordination of lanthanide ions to the phosphorus centers and thus result in the changes of molecular symmetry.

**Table 1:** A comparison of the IR Spectra for compounds **1**–**8**, (CN_3_H_6_)_6_[H_4_P_4_Nb_6_(O_2_)_6_O_24_]∙4H_2_O (P_4_Nb_6_) and Nb_6_.

	*ν*(P–O)	*ν*(O–O)	*ν*(Nb = O), *ν*(Nb–O–Nb)
**1**	1153, 1126, 1091, 1033	846	971, 949, 873, 773, 706, 658, 601, 545
**2**	1154, 1126, 1091, 1033	846	974, 947, 871, 767, 700, 653, 593, 539
**3**	1158, 1126, 1094, 1032	845	970, 947, 871, 765, 698, 649, 597, 546
**4**	1158, 1125, 1090, 1031	846	974, 953, 873, 773, 706, 655, 597, 551
**5**	1156, 1126, 1092, 1033	846	974, 947, 871, 766, 707, 652, 600, 549
**6**	1161, 1133, 1090, 1033	847	973, 946, 873, 766, 701, 655, 596, 545
**7**	1154, 1126, 1091, 1029	846	974, 949, 874, 767, 700, 655, 597, 544
**8**	1160, 1126, 1091, 1029	847	974, 952, 874, 766, 696, 656, 596, 545
**P**_**4**_ **Nb**_**6**_	1157, 1064, 1037	848	997, 974, 947, 876, 765, 658, 558, 527
**Nb**_**6**_	none	none	990, 848, 689, 529

**Figure 5:**
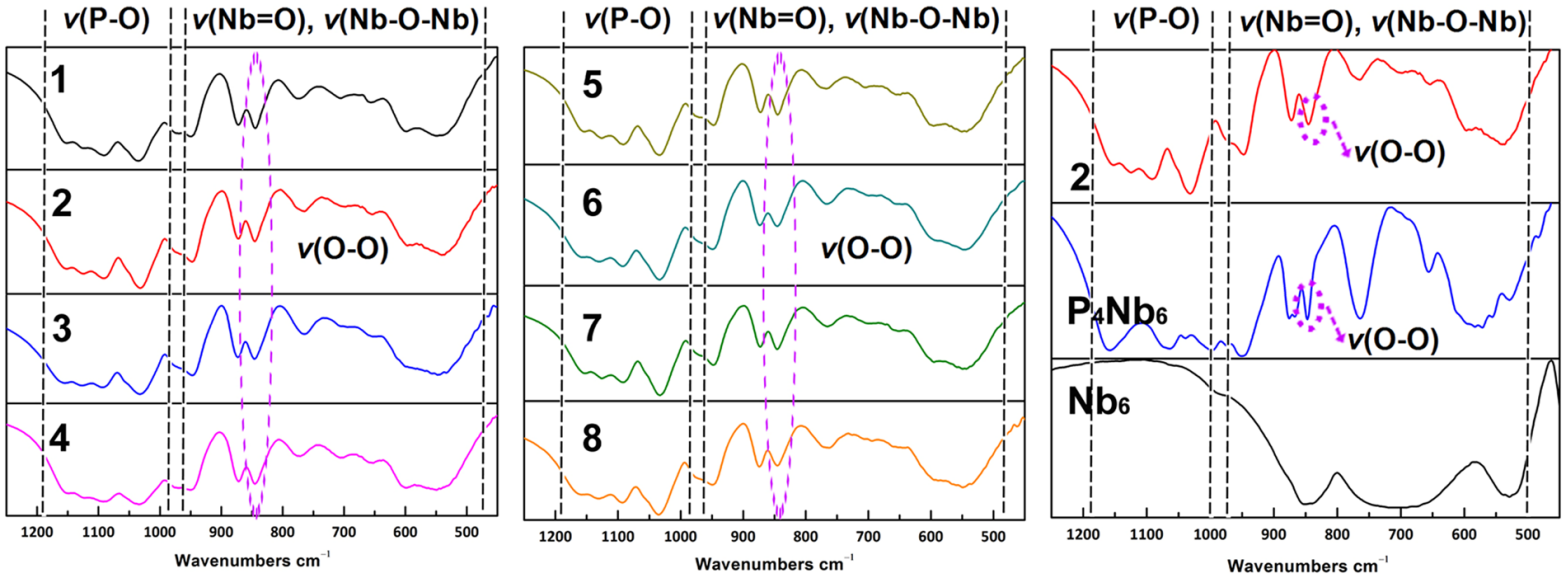
IR spectra of **1**–**8**, (CN_3_H_6_)_6_[H_4_P_4_Nb_6_(O_2_)_6_O_24_]∙4H_2_O and **Nb**_**6**_ in the region between 1250 to 450 cm^−1^.

The significant changes in IR spectra (Fig. 5) of **1**−**8** compared to that of **Nb**_**6**_ are the appearance of strong intensity bands at 850 cm^−1^ and in the region 1200-1000 cm^−1^, which is characteristic of the antisymmetric stretching vibrations of peroxo group^29, 31^ and P–O bond, respectively. This is in satisfactory agreement with the solid-state structure. Additionally, the above-mentioned results confirm that the 6-peroxoniobio-4-phosphate framework formed *in-situ* remains intact under the condition of the synthesis, and further indicate that the developed strategy may be further applicable to molecules of the class of PONb-based lanthanide derivatives.

**Figure 6:**
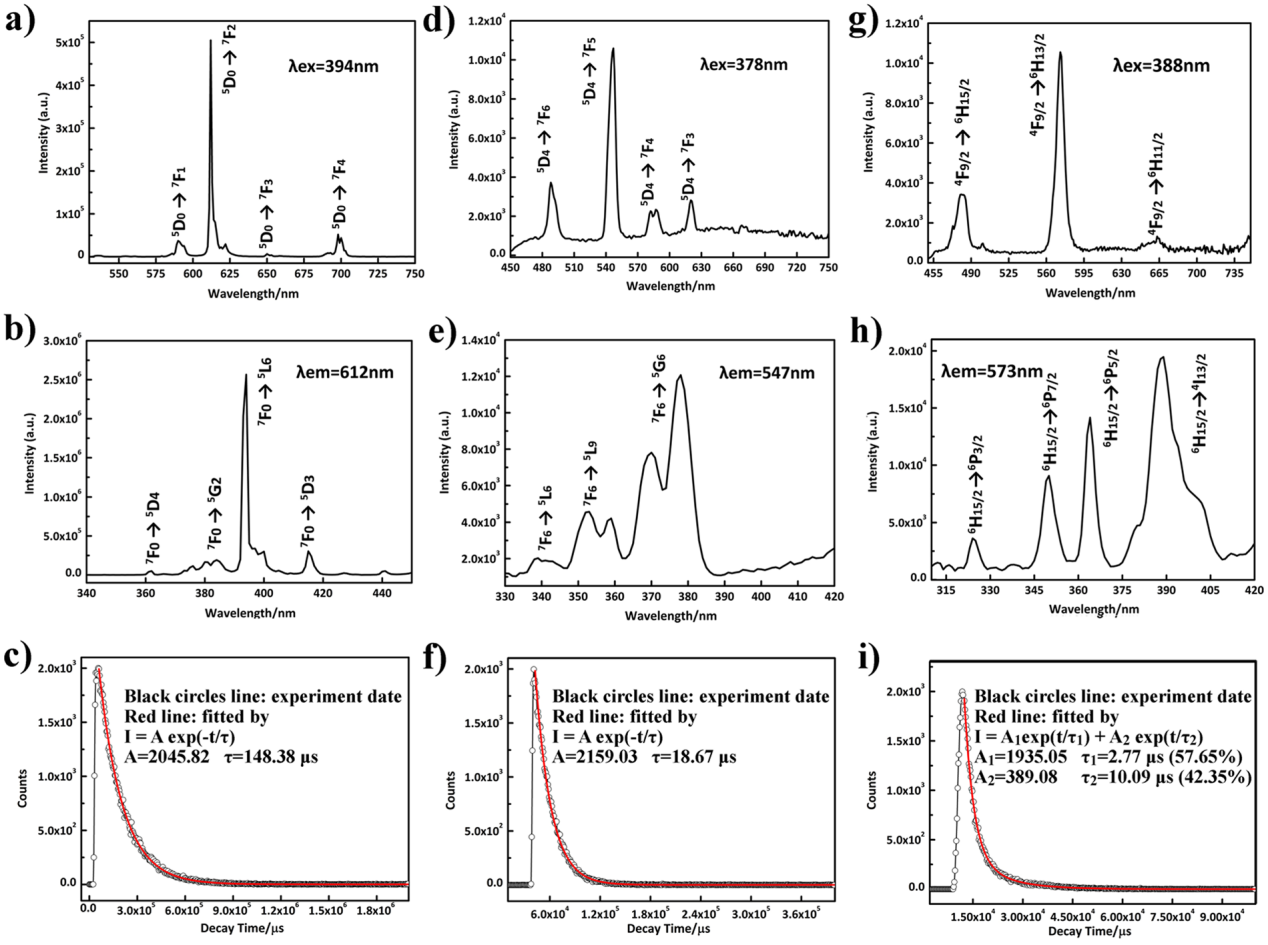
(**a**) The emission spectrum of **1** under excitation at 394 nm at room temperature. (**b**) The emission spectrum of **1** under excitation at 612 nm at room temperature. (**c**) The decay curve of **1**. (**d**) The emission spectrum of **3** under excitation at 378 nm at room temperature. (**e**) The emission spectrum of **3** under excitation at 547 nm at room temperature. (**f**) The decay curve of **3**. (**g**) The emission spectrum of **4** under excitation at 388 nm at room temperature. (**h**) The emission spectrum of **4** under excitation at 573 nm at room temperature. (**i**) The decay curve of **4**.

The photoluminescence behaviours of compounds **1**, **3** and **4** in solid state at room temperature are depicted in Fig. 6, which displays intense photoluminescence upon excitation at 394, 378 and 388 nm for compounds **1**, **3** and **4**, respectively. The emission spectrum of compound **1** exhibits four characteristic emission bands at 590, 612, 653 and 698 nm, corresponding to ^5^D_0_→^7^F_1_,^5^D_0_→^7^F_2_,^5^D_0_→^7^F_3_ and ^5^D_0_→^7^F_4_ transitions of the Eu^3+^ ions (Fig. 6a). These are in good agreement with previous results^39, 48^. The ^5^D_0_→^7^F_1,3_ transitions are magnetic dipole transitions and insensitive to their coordination environments, while ^5^D_0_→^7^F_2,4_ transitions are electric dipole transitions and sensitive to their local environments^49^. The transition at 590 nm belongs to the magnetic dipole ^5^D_0_→^7^F_1_ transition and its emission intensity scarcely varies with the strength of the ligand field exerted on the Eu^3+^ ions, whereas the highest relative intensity of the ^5^D_0_→^7^F_2_ transition at 612 nm is the electric dipole transition and implies red emission light of **1**. Further, the intensity of ^5^D_0_→^7^F_2_ transition is extremely sensitive to chemical bonds in the vicinity of the Eu^3+^ ions. The ^5^D_0_→^7^F_2_/^5^D_0_→^7^F_1_ ratio is widely regarded as a measured of the coordination state and site symmetry of the lanthanide^50^. However, it should be noted that this ratio is easily influenced by other factors such as the polarizability of the ligands. For **1**, the value is ca. 13.5 implying the low site symmetry of the Eu^3+^ ions, which agrees well with the distorted dodecahedral geometry of Eu^3+^ ions in **1**. Furthermore, the excitation spectrum of **1** monitored at the Eu^3+ 5^D_0_→^7^F_2_ transition (612 nm) contains a narrow band and several weak bands (Fig. 6b). The narrow band at 394 nm is attributed to the ^7^F_0_→^5^L_6_ transition of the intra-4f^6^, the three weak peaks in the range of 300–445 nm are assigned to ^7^F_0_→^5^D_4_ (362 nm), ^7^F_0_→^5^G_2_ (385 nm), ^7^F_0_→^5^D_3_ (416 nm) transition, respectively^51^. In order to obtain the lifetime, the luminescence decay curve of **1** was measured at room temperature by monitoring the strongest emission of ^5^D_0_→^7^F_2_ (Figs 6c and S4), which can be fitted successfully to a single exponential function as the equation *I* = *A* exp(−*t*/*τ*). The affording lifetime (*τ*) is 148.38 μs with a pre-exponential factor (A) of 2045.82.

Furthermore, the emission spectrum of **3** upon excitation at 378 nm exhibits a maximum at 546 nm corresponding to the ^5^D_4_→^7^F_5_ transition of Tb^3+^ ion, while the peaks located at 488, 588 and 620 nm are attributed to the ^5^D_4_→^7^F_6_, ^5^D_4_→^7^F_4_ and ^5^D_4_→^7^F_3_ transitions of Tb^3+^ ion (Fig. 6d), respectively^52–54^. Interestingly, the excitation spectrum of **3** upon the excitation at 546 nm consists of three dominant emission bands at 343, 353 and 378 nm, which can be ascribed to the ^7^F_6_→^5^L_6_, ^7^F_6_→^5^L_9_ and ^7^F_6_→^5^G_6_ transitions (Fig. 6e), respectively^55^. The luminescence lifetime of **3** was monitored and can also conform to a single exponential function with a lifetime 18.67 μs (Figs 6f and S5). Meanwhile, the emission spectrum of **4** under excitation at 388 nm displays one high-intensity emission peak at 573 nm and two low-intensity emission peaks at 481 and 663 nm, which is assigned to the ^4^F_9/2_→^6^H_13/2_, ^4^F_9/2_→^6^H_15/2_ and ^4^F_9/2_→^6^H_11/2_ transitions of Dy^3+^ ions (Fig. 6g), respectively^56^. It is noteworthy that the intensity of the^4^F_9/2_→^9^H_13/2_ electric dipole transition is much higher than that of the ^4^F_9/2_→^6^H_15/2_ magnetic dipole transition, illustrating that the Dy^3+^ ions reside in low symmetrical environments without inversion. The excitation spectrum of **4** collected by monitoring the emission at 573 nm is presented in Fig. 6h, and the most intense peak is observed at 388 nm (^6^H_15/2_→^4^I_13/2_), whereas the other three relatively weak peaks are located at 324 nm (^6^H_15/2_→^6^P_3/2_), 350 nm (^6^H_15/2_→^6^P_7/2_) and 364 nm (^6^H_15/2_→^6^P_5/2_), respectively. Interestingly, the lifetime curve for **4** can be well-fitted using a second-order exponential function *I* = *A*_1_ exp(*t*/*τ*_1_) + *A*_2_ exp(*t*/*τ*_2_), affording the luminescence lifetimes *τ*_1_ and *τ*_2_ as 2.77 μs (57.65%) and 10.09 μs (42.35%) (Figs 6i and S6), respectively. The average lifetime τ* is calculated as 5.87 μs based on the formula τ* = (A_1_τ_1_^2^ + A_2_τ_2_^2^)/(A_1_τ_1_ + A_2_τ_2_)^57^.

**Figure 7:**
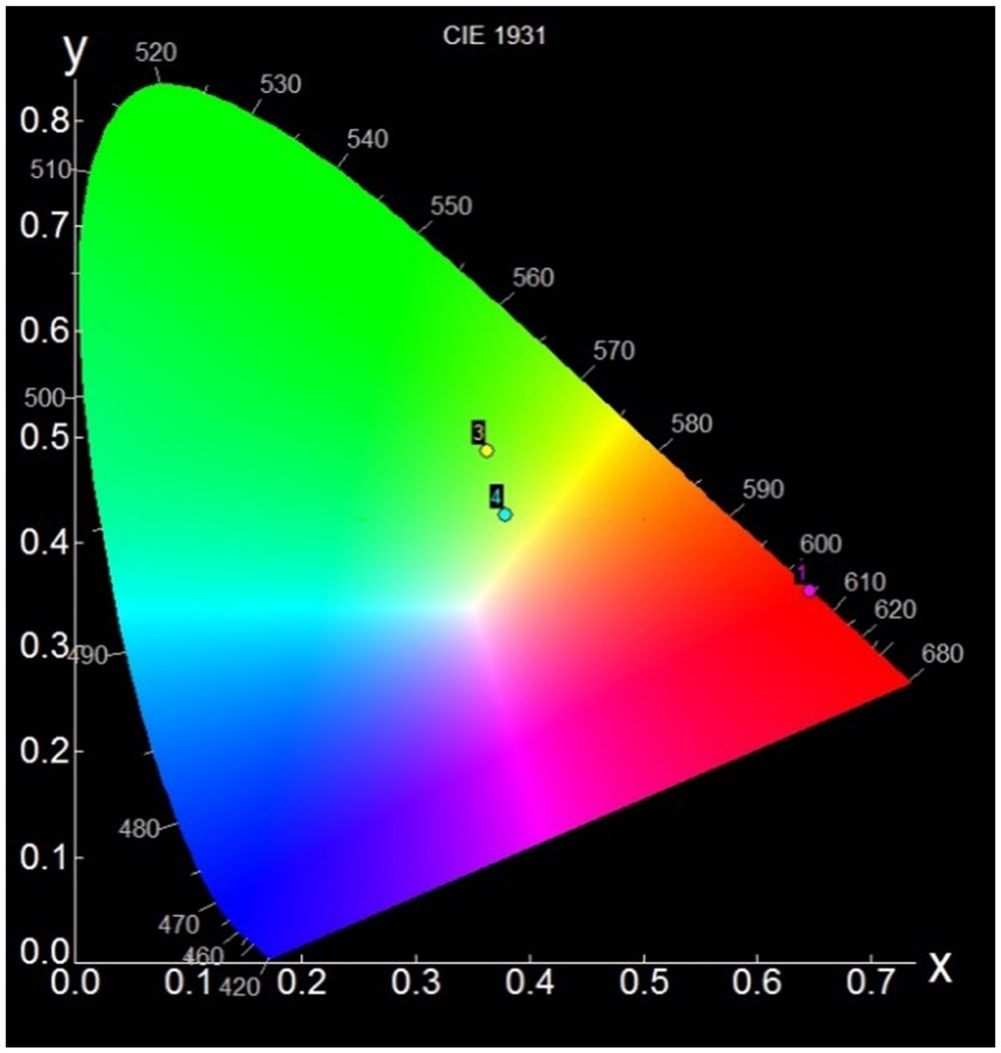
CIE diagram of overall emission of **1**, **3** and **4**.

The flat, tongue-shaped CIE chromatic diagram represents an internationally agreed method of color identification by combining three primary colors (red, green and blue), which will be seen in light with a wavelength. And with a certain conversion, it is important that only two new values (x and y) can be shown on a two-dimensional chart, where x and y represents the horizontal and vertical axis, respectively. In order to name colors, the emission spectra of **1**, **3** and **4** were converted into the x and y coordinates in the CIE chromatic diagram (Fig. 7). The (x, y) values for **1**, **3** and **4** are found to be (0.65, 0.35), (0.36, 0.48) and (0.38, 0.43), respectively, corresponding the reddish orange for **1**, green for **3** and yellowish green for **4**.

## Conclusion

In summary, we have developed a new synthetic method for the synthesis of a new family of PONb-based lanthanoid complexes, **1**–**8**. In order to accomplish the synthesis, a new strategy was developed and successfully applied. The experimental aspects of the strategy include *in-situ* formation of **P**_**4**_(**NbO**_**2**_ **)**_**6**_ building block in acidic media, addition of lanthanide chloride salt and pH adjustment to the desired value. All compounds have been fully characterized in the solid state by single-crystal X-ray diffraction, IR spectra, TG-MS, PXRD. Moreover, their luminescence and lifetime decay behaviors were also investigated systematically. This study not only enriches the structural diversity of lanthanide derivatives containing PONb aggregates, but also provides a convenient synthetic route to PONb-based lanthanoid clusters. In future work, we will extend this approach to isolate various symmetries with the aim of providing *in-situ* formed building block that can be used to design and systematically tailor new luminescent materials with control.

## Experimental Section

### Materials and methods

All the reagents were obtained from commercial sources and used as received. All solvents were used without further purification. K_7_H[Nb_6_O_19_]·13H_2_O^58^ were prepared using literature methods.

#### Synthesis of 1

K_7_H[Nb_6_O_19_]·13H_2_O (0.55 g, 0.40 mmol) was dissolved in a solution consisting of 4.0 mL H_2_O_2_ (30%) and 36 mL of water. Under rapidly stirring, 1.0 mL H_3_PO_4_ (3.0 M) was added. Twenty minutes later (at this point pH was about 2.5), a solution of EuCl_3_·6H_2_O (1.47 g, 4.0 mmol in 2 mL H_2_O) was added. The resultant solution was adjusted to pH 1.53 and heated to 90 °C for 4 h. Then, the mixture was cooled to room temperature and filtered. The clear filtrate was kept at room temperature to allow slow evaporation. Subsequent crystallization over 2 weeks yielded a bright-yellow hydrated salt in 12% yield (based on Nb).

#### Synthesis of 2–8

Employing GdCl_3_·6H_2_O (1.49 g, 4.0 mmol), TbCl_3_·6H_2_O (1.50 g, 4.0 mmol), DyCl_3_·6H_2_O (1.48 g, 4.0 mmol), HoCl_3_·6H_2_O (1.53 g, 4.0 mmol), ErCl_3_·6H_2_O (1.52 g, 4.0 mmol), TmCl_3_·7H_2_O (1.61 g, 4.0 mmol), YbCl_3_·6H_2_O (1.53 g, 4.0 mmol) instead of EuCl_3_·6H_2_O (1.47 g, 4.0 mmol) under similar reaction conditions, with the pH value adjusted to 1.96, 1.82, 1.58, 1.74, 1.72, 1.25 and 1.26 for **2**–**8**, respectively. Average yields are 10–20% based on niobium.

### Physical measurements

IR spectra (ν = 4000–400 cm^−1^) of the samples were recorded on a PerkinElmer FT-IR spectrometer using KBr pellets. Powder X-ray diffraction (PXRD) data were recorded on a Bruker D8 Advance instrument with Cu Kα radiation (λ = 1.5418 Å) in the angular range 2θ = 5–50° at 293 K. Thermogravimetric analyses (TGA) were measured on a NETZSCH STA 449 F5 Jupiter thermal analyzer in flowing N_2_ with a heating rate of 10 °C·min^−1^. Photoluminescence properties were performed on EDINBURGH FLS 980 fluorescence spectrophotometer.

### X-ray crystallography

Suitable single crystals of **1**–**8** were selected from their respective mother liquors and placed in a thin glass tube. X-ray diffraction intensity was recorded on a Bruker Apex-II CCD diffractometer at 296(2) K with MoKa monochromated radiation (λ = 0.71073 Å). Structure solution and refinement were carried out by using the SHELXS-97 and SHELXL-2014 program package^59, 60^ for **1**–**8**. Selected details of the data collection and structural refinement of compounds **1**–**8** can be found in Table S5. Further details of the crystal structure investigation can be obtained from the Fachinformationszentrum Karlsruhe, 76344 Eggenstein-Leopoldshafen, Germany (fax: (+49)7247–808–666; e-mail: crysdata@fiz-karlsruhe.de) on quoting the depository CSD numbers 432370–432377 for **1**–**8**.

## Electronic supplementary material

Supplementary Information
