# Supplementary material for: Synthesis, structure, and luminescent properties of a family of lanthanide-functionalized peroxoniobiophosphates
Source: Sci Rep. 2017 Sep 6;7:10653. doi: 10.1038/s41598-017-10811-2 (PMC5587641; doi:10.1038/s41598-017-10811-2)
Supplement: Supplementary file 1 — Supplementary Information [file 41598_2017_10811_MOESM1_ESM.pdf]

Supplementary Information

Synthesis, structure, and luminescent properties of a family of lanthanide-functionalized peroxoniobiophosphates

Haiying Wang, Jing Li, Junjun Sun, Yaya Wang, Zhijie Liang, Pengtao Ma, Dongdi Zhang,* Jingping Wang and Jingyang Niu*

Henan Key Laboratory of Polyoxometalate Chemistry, College of Chemistry and Chemical Engineering, Henan University, Kaifeng, Henan 475004, P. R. China.

*Correspondence and requests for materials should be addressed to D.Z. (E-mail: ddzhang@henu.edu.cn) and J.N. (E-mail: niujy@henu.edu.cn)

CONTENTS

1. Table S1. Survey of the reported polyoxoniobate-based rare-earth clusters.
2. Table S2. Bond length in **1−8**.
3. Table S3. BVS calculation results for **1−8**.
4. Table S4. O_p–Nb–O_p angle (°) in **1−8**.
5. Table S5. Crystallographic data and structure refinements for **1−8**.
6. Figure S1. Ball-and-stick representation of peroxo-$\{Nb_6\}$ and P_2Nb_3.
7. Figure S2. IR spectra of compound **2**, $\mathbf{P_4Nb_6}$ and $K_7H[Nb_6O_{19}]\cdot 13H_2O$ in the region between 4000 to 450 cm^{-1}.
8. Figure S3. IR spectra of **1−8** in the region between 4000 to 450 cm^{-1}.
9. Figure S4. The decay curve of **1**.
10. Figure S5. The decay curve of **3**.
11. Figure S6. The decay curve of **4**.
12. Figure S7. Photos of luminescent compounds **1**, **3** and **4** under UV light (365 nm).
13. Figure S8. The comparison of experimental and simulated XRPD patterns of **1−8**.
14. Figure S9. The TG-MS curves of **1−8**.

15. Figure S10. Representations of structural figure with probability ellipsoids of **1−8**.

Table S1. Survey of the reported Polyoxoniobate-based rare-earth clusters.

Polyanion	RE	Ref
$\{[Eu_3O(OH)_3(OH_2)_3]_2A_{12}(Nb_6O_{19})_5\}$	Eu^{3+}	Yamase et al. (1994) [1]
$[\{Er_{30}(OH)_3(H_{20})_3\}_2Al_2(Nb_60_{19})_5]$	Er^{3+}	Naruke and Yamase (1996) [2]
$[\{Lu_3O(OH)_3(H_2O)_3\}_2Al_2(Nb_6O_{19})_5]$	Lu^{3+}	Naruke and Yamase (1997) [3]
$[Tb_{4.3}Eu_{1.7}O_2(OH)_6(H_2O)_6Al_2(Nb_6O_{19})_5]$		Yamase et al. (1999) [4]
$[Ln_6(H_2O)_{38}[P_2W_{15}Nb_3O_{62})_4]^{18-}$	Ce^{3+}, Eu^{3+}	Liu et al. (2012) [5]
$[(Ge_2W_{18}Nb_6O_{78})Eu(H_2O)_4]^{7-}$		
$[(Ge_4W_{36}Nb_{12}O_{156})Eu(H_2O)_3]^{17-}$		
$[Cs(GeW_9Nb_3O_{40})_4(SO_4)Eu_5(H_2O)_{36}]^{14-}$		
$[Cs_2(GeW_9Nb_3O_{40})_4Eu_4(H_2O)_{22}]^{14-}$	Eu^{3+}	Liu et al. (2012) [6]
$[(GeW_9Nb_3O_{40})_4Eu_{5.5}(H_2O)_{26}]^{11.5-}$		
$[GeW_9Nb_3O_{40}Eu_{1.25}(H_2O)_{12}]^{3.25-}$		
$\{Ln_{12}W_{12}O_{36}(H_2O)_{24}(Nb_6O_{19})_{12}\}$	(Ln = Y, La, Sm, Eu, Yb)	Zheng et al. (2016) [7]

Table S2. Bond length in **1−8**.

Nb–O distance (Å)								
	1	2	3	4	5	6	7	8
Nb1–O2	2.071(5)	2.075(5)	2.070(6)	2.072(7)	2.071(5)	2.067(5)	2.065(5)	2.076(5)
Nb1–O3	2.098(5)	2.099(6)	2.101(6)	2.102(7)	2.096(5)	2.090(6)	2.098(6)	2.095(5)
Nb1–O10	1.863(5)	1.856(6)	1.864(7)	1.864(8)	1.865(5)	1.824(6)	1.882(6)	1.873(5)
Nb1–O11	2.038(6)	2.044(6)	2.040(7)	2.038(7)	2.041(5)	2.053(6)	2.036(6)	2.039(5)
Nb1–O12	2.106(5)	2.105(6)	2.098(6)	2.097(8)	2.104(5)	2.154(5)	2.096(6)	2.097(5)
Nb1–O14	1.981(6)	1.986(6)	1.973(7)	1.974(8)	1.982(6)	1.991(6)	1.982(6)	1.982(5)
Nb1–O16	1.966(6)	1.971(6)	1.967(7)	1.977(9)	1.968(6)	1.981(6)	1.969(6)	1.965(6)
Nb2–O2	2.057(5)	2.057(5)	2.059(6)	2.063(7)	2.063(5)	2.029(5)	2.089(5)	2.066(5)
Nb2–O3	2.047(5)	2.052(6)	2.046(6)	2.042(8)	2.046(5)	2.052(6)	2.039(6)	2.049(5)
Nb2–O5	2.024(6)	2.026(6)	2.016(7)	2.023(8)	2.021(6)	2.030(6)	2.008(6)	2.026(6)

Nb2–O7	2.029(5)	2.027(6)	2.020(7)	2.022(7)	2.029(5)	2.035(6)	2.030(6)	2.024(5)
Nb2–O8	1.954(6)	1.956(6)	1.952(7)	1.944(8)	1.952(6)	1.958(6)	1.960(6)	1.949(6)
Nb2–O9	1.965(6)	1.970(7)	1.967(8)	1.970(8)	1.959(7)	1.985(6)	1.940(8)	1.970(6)
Nb2–O15	1.969(7)	1.971(7)	1.969(8)	1.979(9)	1.966(7)	1.980(6)	1.957(8)	1.967(6)
Nb3–O1	2.043(5)	2.043(6)	2.044(6)	2.048(7)	2.044(5)	2.050(5)	2.049(6)	2.044(5)
Nb3–O2	2.093(5)	2.090(5)	2.089(6)	2.085(7)	2.086(5)	2.091(5)	2.079(5)	2.086(5)
Nb3–O5	2.017(6)	2.009(6)	2.032(7)	2.033(8)	2.017(6)	1.915(6)	2.048(6)	2.036(6)
Nb3–O10	1.948(5)	1.960(6)	1.938(7)	1.939(8)	1.945(5)	2.008(6)	1.925(6)	1.934(5)
Nb3–O13	2.110(5)	2.114(6)	2.102(7)	2.123(7)	2.110(5)	2.120(6)	2.111(6)	2.108(5)
Nb3–O17	1.930(9)	1.930(10)	1.923(10)	1.920(11)	1.938(9)	1.970(8)	1.942(8)	1.938(8)
Nb3–O18	1.912(9)	1.903(11)	1.916(10)	1.918(12)	1.913(9)	1.988(8)	1.921(8)	1.918(8)

Ln–O distance (Å)

	**1**	**2**	**3**	**4**	**5**	**6**	**7**	**8**
O4	2.275(6)	2.268(6)	2.243(7)	2.227(8)	2.231(6)	2.215(6)	2.205(6)	2.197(6)
O6	2.306(5)	2.302(6)	2.291(7)	2.267(8)	2.265(6)	2.252(6)	2.239(6)	2.237(5)
O1W	2.399(7)	2.391(7)	2.368(8)	2.368(10)	2.345(7)	2.363(7)	2.322(8)	2.308(7)
O2W	2.471(7)	2.455(7)	2.436(8)	2.427(9)	2.419(7)	2.413(8)	2.395(7)	2.383(6)
O3W	2.488(7)	2.484(7)	2.468(8)	2.449(10)	2.438(7)	2.422(7)	2.418(7)	2.409(7)
O4W	2.473(7)	2.454(7)	2.450(8)	2.441(9)	2.418(7)	2.382(7)	2.405(7)	2.395(6)
O5W	2.436(8)	2.424(8)	2.421(9)	2.408(11)	2.387(8)	2.374(9)	2.373(8)	2.362(7)
O6W	2.504(7)	2.491(7)	2.478(8)	2.472(9)	2.455(6)	2.465(8)	2.443(7)	2.435(6)

P–O distance (Å)

	**1**	**2**	**3**	**4**	**5**	**6**	**7**	**8**
P1–O1	1.533(6)	1.537(6)	1.521(7)	1.519(8)	1.531(6)	1.535(6)	1.530(6)	1.533(6)
P1–O4	1.496(6)	1.499(6)	1.498(7)	1.507(8)	1.496(6)	1.502(6)	1.500(6)	1.495(6)
P1–O8	1.547(6)	1.547(6)	1.544(7)	1.553(8)	1.548(6)	1.546(6)	1.546(6)	1.552(6)
P1–O11	1.525(6)	1.522(6)	1.520(7)	1.524(8)	1.521(6)	1.524(6)	1.527(6)	1.522(6)
P2–O6	1.503(6)	1.503(6)	1.488(7)	1.501(8)	1.499(6)	1.503(6)	1.497(6)	1.497(6)
P2–O7	1.562(6)	1.566(6)	1.566(7)	1.570(7)	1.561(6)	1.569(6)	1.564(6)	1.565(5)
P2–O12	1.542(5)	1.548(6)	1.540(7)	1.548(8)	1.544(5)	1.538(6)	1.551(6)	1.546(5)
P2–O13	1.552(6)	1.550(6)	1.553(7)	1.539(8)	1.550(6)	1.552(6)	1.550(6)	1.550(5)

O–O distance (Å)

	**1**	**2**	**3**	**4**	**5**	**6**	**7**	**8**

O9–O15	1.491(9)	1.484(10)	1.490(11)	1.490(13)	1.481(9)	1.503(9)	1.432(11)	1.494(9)
O14–O16	1.480(8)	1.478(8)	1.480(9)	1.490(11)	1.476(8)	1.479(8)	1.467(9)	1.483(8)
O17–O18	1.389(12)	1.355(14)	1.419(13)	1.420(15)	1.396(12)	1.447(11)	1.442(11)	1.425(10)

Table S3. BVS calculation results for **1−8**.

Polyanion **1**					
Atom Code	Value	Atom Code	Value	Atom Code	Value
Eu	3.23	O3	1.30	O11	1.99
P1	5.13	O4	1.97	O12	1.82
P2	4.94	O5	1.49	O13	1.78
Nb1	5.34	O6	1.90	O14	0.83
Nb2	5.44	O7	1.89	O15	0.86
Nb3	5.50	O8	2.10	O16	0.86
O1	1.95	O9	0.86	O17	0.95
O2	1.93	O10	2.04	O18	0.996
Polyanion **2**					
Atom Code	Value	Atom Code	Value	Atom Code	Value
Gd	3.24	O3	1.29	O11	1.99
P1	5.12	O4	1.96	O12	1.80
P2	4.91	O5	1.50	O13	1.78
Nb1	5.36	O6	1.89	O14	0.82
Nb2	5.41	O7	1.88	O15	0.85
Nb3	5.51	O8	2.09	O16	0.85
O1	1.94	O9	0.85	O17	0.95
O2	1.93	O10	2.04	O18	1.02
Polyanion **3**					
Atom Code	Value	Atom Code	Value	Atom Code	Value
Tb	3.09	O3	1.29	O11	2.01
P1	5.19	O4	1.94	O12	1.84
P2	4.99	O5	1.47	O13	1.79
Nb1	5.40	O6	1.92	O14	0.85
Nb2	5.47	O7	1.89	O15	0.86
Nb3	5.52	O8	2.11	O16	0.86
O1	2.00	O9	0.86	O17	0.97

O2	1.94	O10	2.07	O18	0.99

Polyanion **4**

Atom Code	Value	Atom Code	Value	Atom Code	Value
Dy	2.94	O3	1.30	O11	2.00
P1	5.12	O4	1.89	O12	1.81
P2	4.94	O5	1.46	O13	1.80
Nb1	5.37	O6	1.86	O14	0.84
Nb2	5.44	O7	1.88	O15	0.83
Nb3	5.48	O8	2.10	O16	0.84
O1	1.99	O9	0.85	O17	0.97
O2	1.94	O10	2.06	O18	0.98

Polyanion **5**

Atom Code	Value	Atom Code	Value	Atom Code	Value
Ho	3.23	O3	1.30	O11	2.01
P1	5.14	O4	1.96	O12	1.82
P2	4.96	O5	1.49	O13	1.79
Nb1	5.38	O6	1.90	O14	0.83
Nb2	5.46	O7	1.89	O15	0.86
Nb3	5.49	O8	2.10	O16	0.86
O1	1.96	O9	0.88	O17	0.93
O2	1.94	O10	2.04	O18	0.99

Polyanion **6**

Atom Code	Value	Atom Code	Value	Atom Code	Value
Er	3.00	O3	1.30	O11	1.97
P1	5.11	O4	1.91	O12	1.76
P2	4.93	O5	1.72	O13	1.76
Nb1	5.37	O6	1.85	O14	0.81
Nb2	5.38	O7	1.85	O15	0.83
Nb3	5.30	O8	2.09	O16	0.83
O1	1.94	O9	0.82	O17	0.85
O2	2.00	O10	2.03	O18	0.81

Polyanion **7**

Atom Code	Value	Atom Code	Value	Atom Code	Value
Tm	3.19	O3	1.31	O11	1.99

P1	5.13	O4	1.95	O12	1.80
P2	4.93	O5	1.46	O13	1.78
Nb1	5.34	O6	1.91	O14	0.83
Nb2	5.50	O7	1.88	O15	0.88
Nb3	5.45	O8	2.09	O16	0.85
O1	1.96	O9	0.92	O17	0.92
O2	1.91	O10	2.05	O18	0.97

Polyanion **8**

Atom Code	Value	Atom Code	Value	Atom Code	Value
Yb	2.98	O3	1.30	O11	2.00
P1	5.12	O4	1.92	O12	1.82
P2	4.95	O5	1.45	O13	1.79
Nb1	5.36	O6	1.87	O14	0.82
Nb2	5.43	O7	1.89	O15	0.86
Nb3	5.47	O8	2.10	O16	0.86
O1	1.95	O9	0.85	O17	0.93
O2	1.92	O10	2.05	O18	0.98

Table S4. O_p–Nb–O_p angle (°) in **1−8**.

	**1**	**2**	**3**	**4**	**5**	**6**	**7**	**8**
O16–Nb1–O14	44.0(2)	43.9(2)	44.1(3)	44.3(3)	43.9(2)	43.7(2)	43.6(2)	44.1(2)
O9–Nb2–O15	44.5(3)	44.3(3)	44.5(3)	44.3(4)	44.3(3)	44.5(3)	43.1(3)	44.6(3)
O18–Nb3–O17	42.4(4)	41.4(4)	43.4(4)	43.4(5)	42.5(4)	42.9(3)	43.8(3)	43.4(3)

Table S5. Crystallographic Data and Structure Refinements for **1−8**.

	**1**	**2**	**3**	**4**
Empirical formula	$Eu_2H_{48}P_4Nb_6O_{60}$	$Gd_2H_{48}P_4Nb_6O_{60}$	$Tb_2H_{48}P_4Nb_6O_{60}$	$Dy_2H_{48}P_4Nb_6O_{60}$
Formula weight	1993.64	2004.22	2007.56	2014.72
Temperature/K	296.15	296.15	296.15	296.15
Crystal system	orthorhombic	orthorhombic	orthorhombic	orthorhombic
Space group	*Pbca*	*Pbca*	*Pbca*	*Pbca*
a/Å	14.9750(6)	14.9680(4)	14.945(2)	14.931(3)
b/Å	15.5210(6)	15.5250(4)	15.499(2)	15.490(3)

c/Å	21.1154(9)	21.1221(6)	21.035(3)	21.007(4)
V/Å^{-3}	4907.8(3)	4908.3(2)	4872.3(11)	4858.4(15)
Z	4	4	4	4
ρcalcg/cm^3	2.698	2.712	2.737	2.754
μ/mm $^{-1}$	4.140	4.287	4.499	4.676
F(000)	3840.0	3848.0	3856.0	3864.0
Index ranges	-17 ≤ h ≤ 17	-17 ≤ h ≤ 15	-16 ≤ h ≤ 17	-17 ≤ h ≤ 16
	-18 ≤ k ≤ 11	-18 ≤ k ≤ 11	-18 ≤ k ≤ 17	-18 ≤ k ≤ 11
	-22 ≤ l ≤ 25	-23 ≤ l ≤ 25	-25 ≤ l ≤ 15	-25 ≤ l ≤ 24
Reflections collected	24030	23958	23468	23284
Independent reflections	4356	4364	4330	4319
Rint	0.0244	0.0288	0.0585	0.0785
Data/restraints/parameters	4356/0/175	4364/0/175	4330/0/175	4319/0/175
GOF on F^2	1.044	1.021	1.040	1.026
Final R indexes [I>=2σ (I)][a]	R_1 = 0.0436	R_1 = 0.0448	R_1 = 0.0521	R_1 = 0.0637
	wR_2 = 0.1080	wR_2 = 0.1108	wR_2 = 0.1349	wR_2 = 0.1660
Final R indexes [all data][b]	R_1 = 0.0460	R_1 = 0.0495	R_1 = 0.0604	R_1 = 0.0737
	wR_2 = 0.1098	wR_2 = 0.1143	wR_2 = 0.1440	wR_2 = 0.1795

	**5**	**6**	**7**	**8**
Empirical formula	$Ho_2H_{48}P_4Nb_6O_{60}$	$Er_2H_{42}P_4Nb_6O_{57}$	$Tm_2H_{48}P_4Nb_6O_{60}$	$Yb_2H_{48}P_4Nb_6O_{60}$
Formula weight	2019.58	1970.19	2027.58	2035.80
Temperature/K	296.15	296.15	296.15	296.15
Crystal system	orthorhombic	orthorhombic	orthorhombic	orthorhombic
Space group	*Pbca*	*Pbca*	*Pbca*	*Pbca*
a/Å	14.9045(6)	14.6827(13)	14.8761(8)	14.8795(5)
b/Å	15.4676(6)	15.4255(15)	15.4525(8)	15.4794(5)
c/Å	21.0020(9)	21.1385(19)	20.9356(11)	20.9466(7)
V/Å^{-3}	4841.7(3)	4787.6(8)	4812.5(4)	4824.5(3)
Z	4	4	4	4
ρcalcg/cm^3	2.771	2.733	2.798	2.803
μ/mm $^{-1}$	4.874	5.121	5.303	5.489
F(000)	3872.0	3760.0	3888.0	3896.0
Index ranges	-17 ≤ h ≤ 16	-16 ≤ h ≤ 17	-17 ≤ h ≤ 17	-17 ≤ h ≤ 17
	-18 ≤ k ≤ 12	-18 ≤ k ≤ 16	-11 ≤ k ≤ 18	-17 ≤ k ≤ 18

	-23 ≤ l ≤ 25	-24 ≤ l ≤ 25	-24 ≤ l ≤ 24	-25 ≤ l ≤ 18
Reflections collected	23490	22954	23331	23549
Independent reflections	4291	4250	4279	4283
Rint	0.0229	0.0342	0.0352	0.0264
Data/restraints/parameters	4291/0/175	4250/0/175	4279/0/175	4283/0/175
GOF on F^2	1.040	1.033	1.062	1.042
Final R indexes [I>=2σ (I)][a]	R_1 = 0.0421	R_1 = 0.429	R_1 = 0.0421	R_1 = 0.0384
	wR_2 = 0.1040	wR_2 = 0.1139	wR_2 = 0.1045	wR_2 = 0.0909
Final R indexes [all data][b]	R_1 = 0.0434	R_1 = 0.0452	R_1 = 0.0453	R_1 = 0.0415
	wR_2 = 0.1049	wR_2 = 0.1163	wR_2 = 0.1068	wR_2 = 0.0929

$^aR_1 = \sum||F_o| - |F_c||/\sum|F_o|$. $^bwR_2 = \{\sum[w(F_o^2 - F_c^2)^2]/\sum[w(F_o^2)^2]\}^{1/2}$.

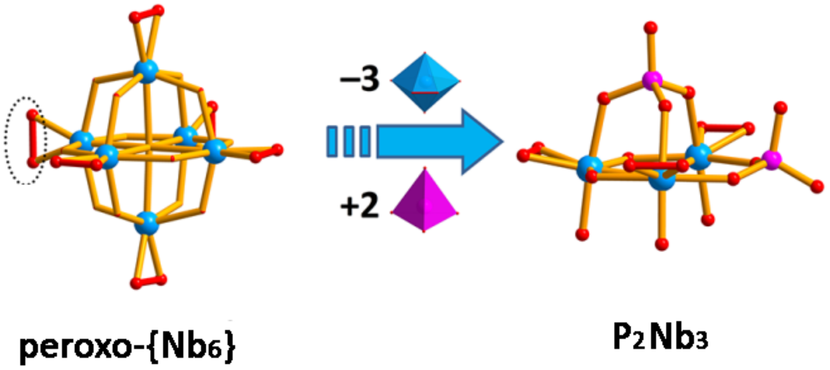

Figure S1. Ball-and-stick representation of peroxo-{Nb_6} and **P_2Nb_3**.

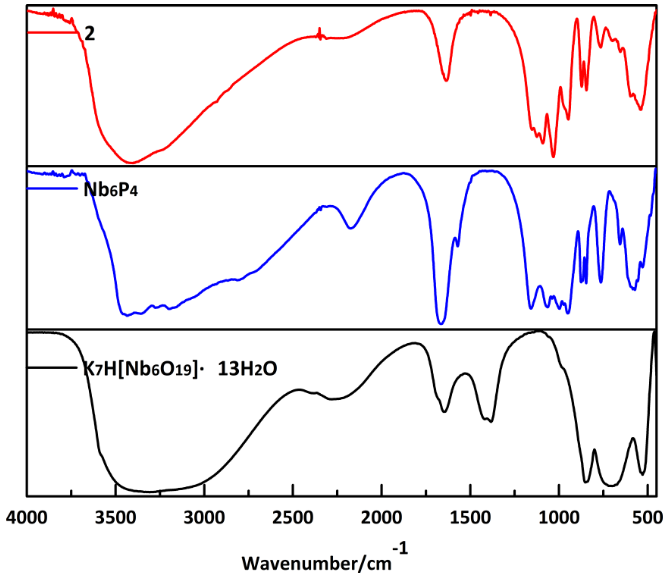

Figure S2. IR spectra of compound **2**, **P_4Nb_6** and $K_7H[Nb_6O_{19}]\cdot 13H_2O$ in the region between 4000 to 450 cm^{-1}.

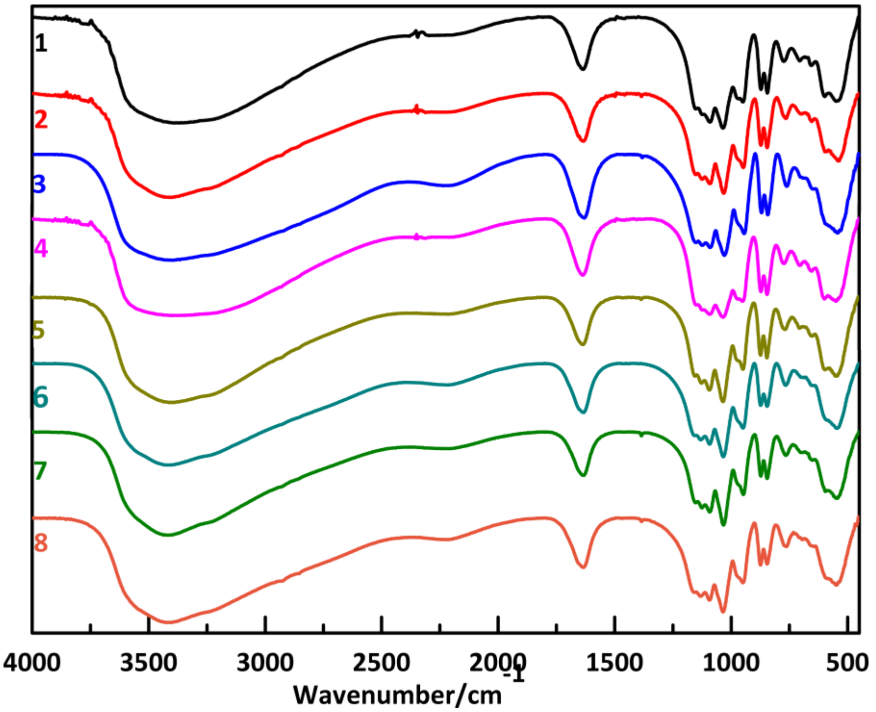

Figure S3. IR spectra of **1−8** in the region between 4000 to 450 cm^{-1}.

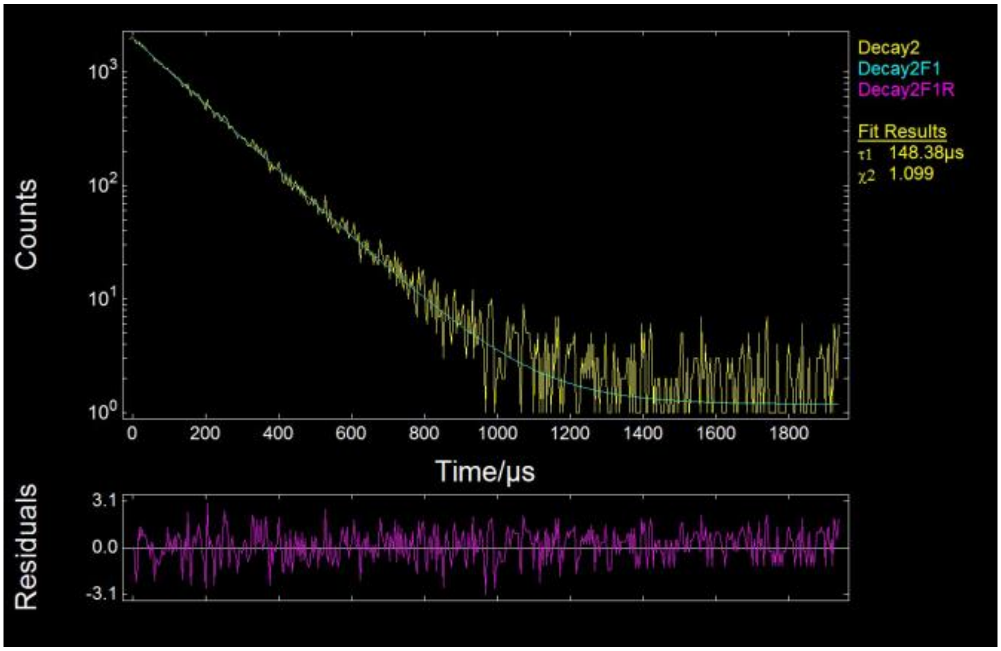

Figure S4. The decay curve of **1**.

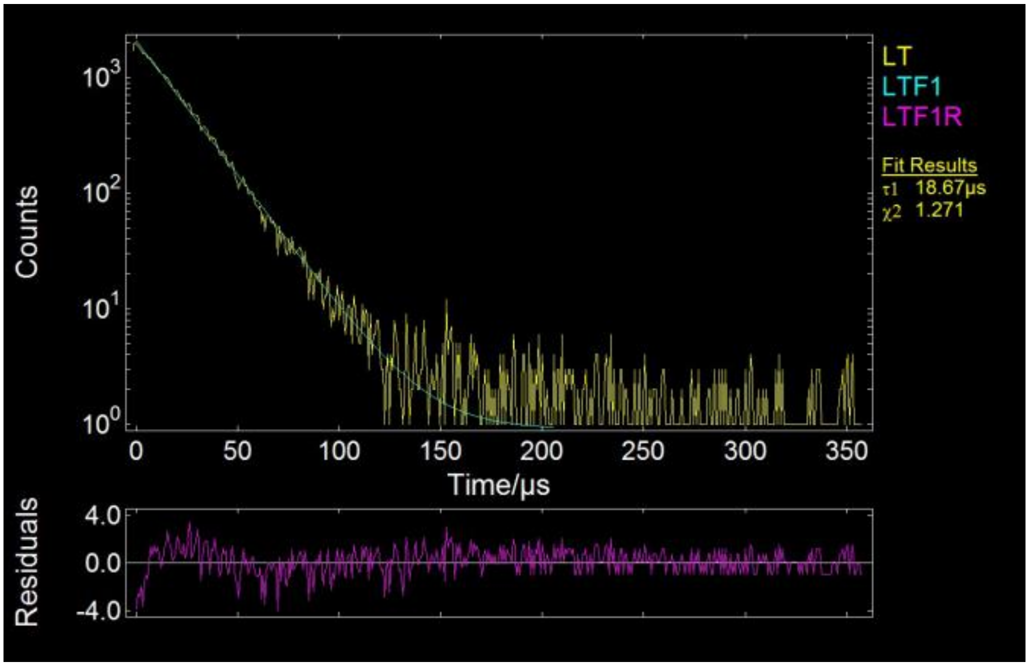

Figure S5. The decay curve of **3**.

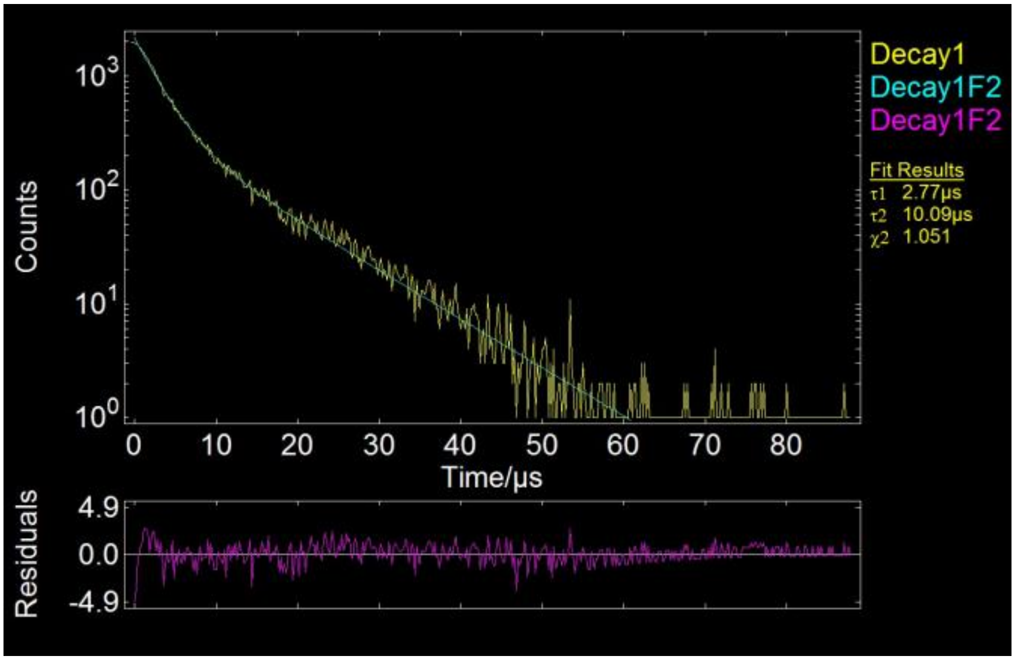

Figure S6. The decay curve of **4**.

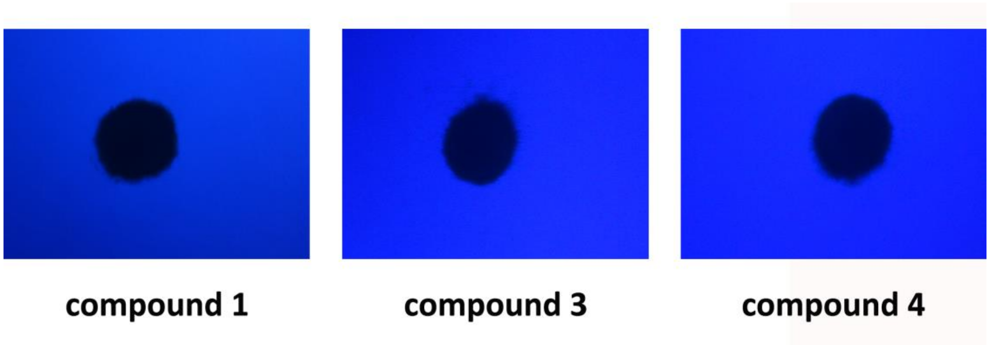

Figure S7. Photos of luminescent compounds **1**, **3** and **4** under UV light (365nm)**.**

As shown in the Figure S7, the luminescent compounds **1**, **3** and **4** were not bright under UV light, or even no light. This may due to the coordination of water. Each of the Ln atoms is eight-coordinated, which is ligated by six terminal aqua ligands and two bridging oxygen atoms. It has nothing to do with Ln-Ln distances. In addition, we had tested the QY of compounds **1**, **3** and **4**. However, the results show that the QY are nearly zero due to the weak emission intensity.

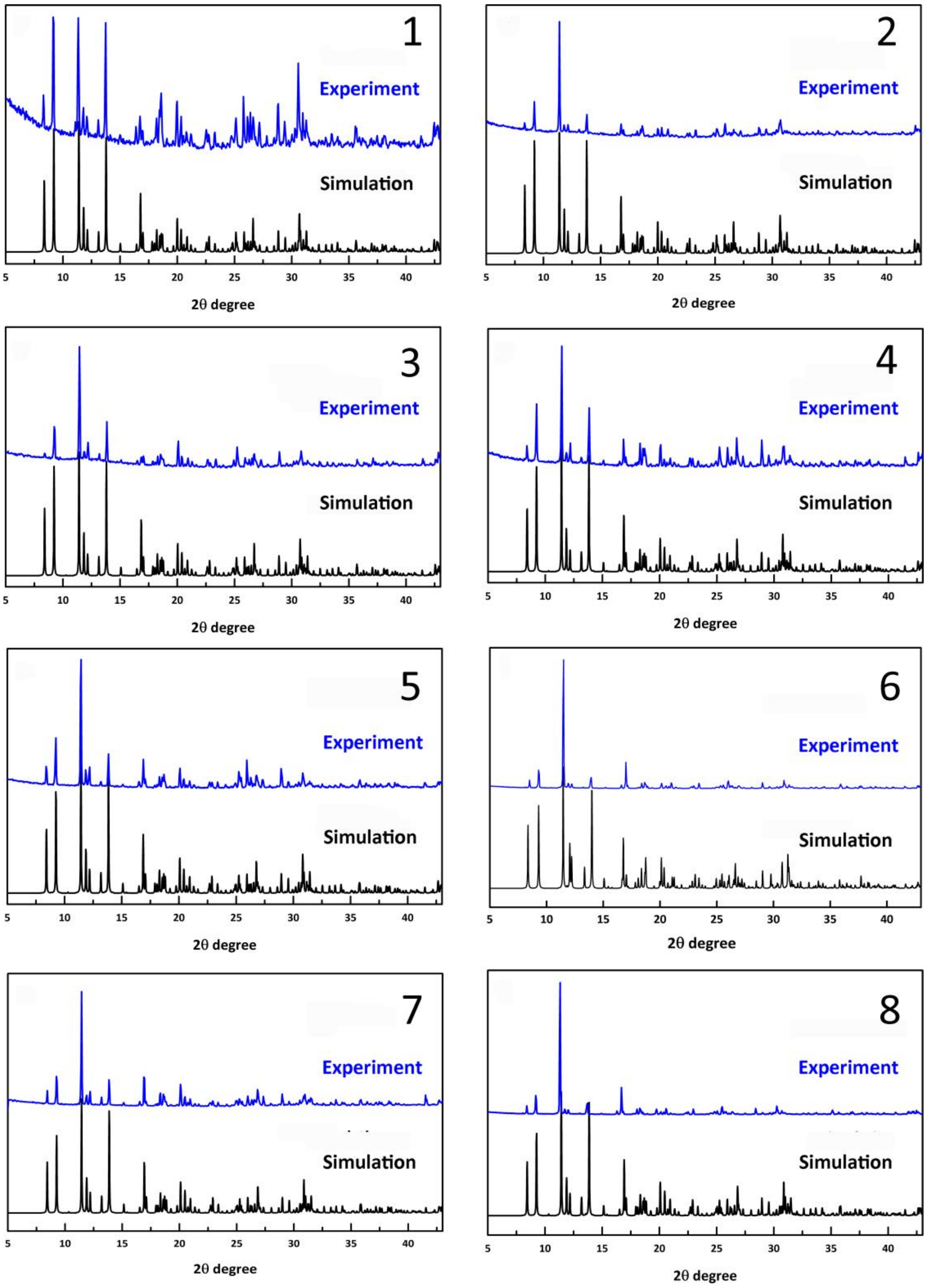

Figure S8. The comparison of experimental (blue) and simulated (black) XRPD patterns of **1−8**.

The peak positions of the simulated and experimental PXRD patterns do not match very well with each other (Figure S8), which is probably due to the solvent loss. On the other hand, the difference in the intensities of some diffraction peaks may be attributed to the preferred orientation of the crystalline powder samples. It should be noted that the sample crystals are

picked out one by one under a microscope, and then washed by cold water. Thus, we think the purity of the complexes can be guaranteed.

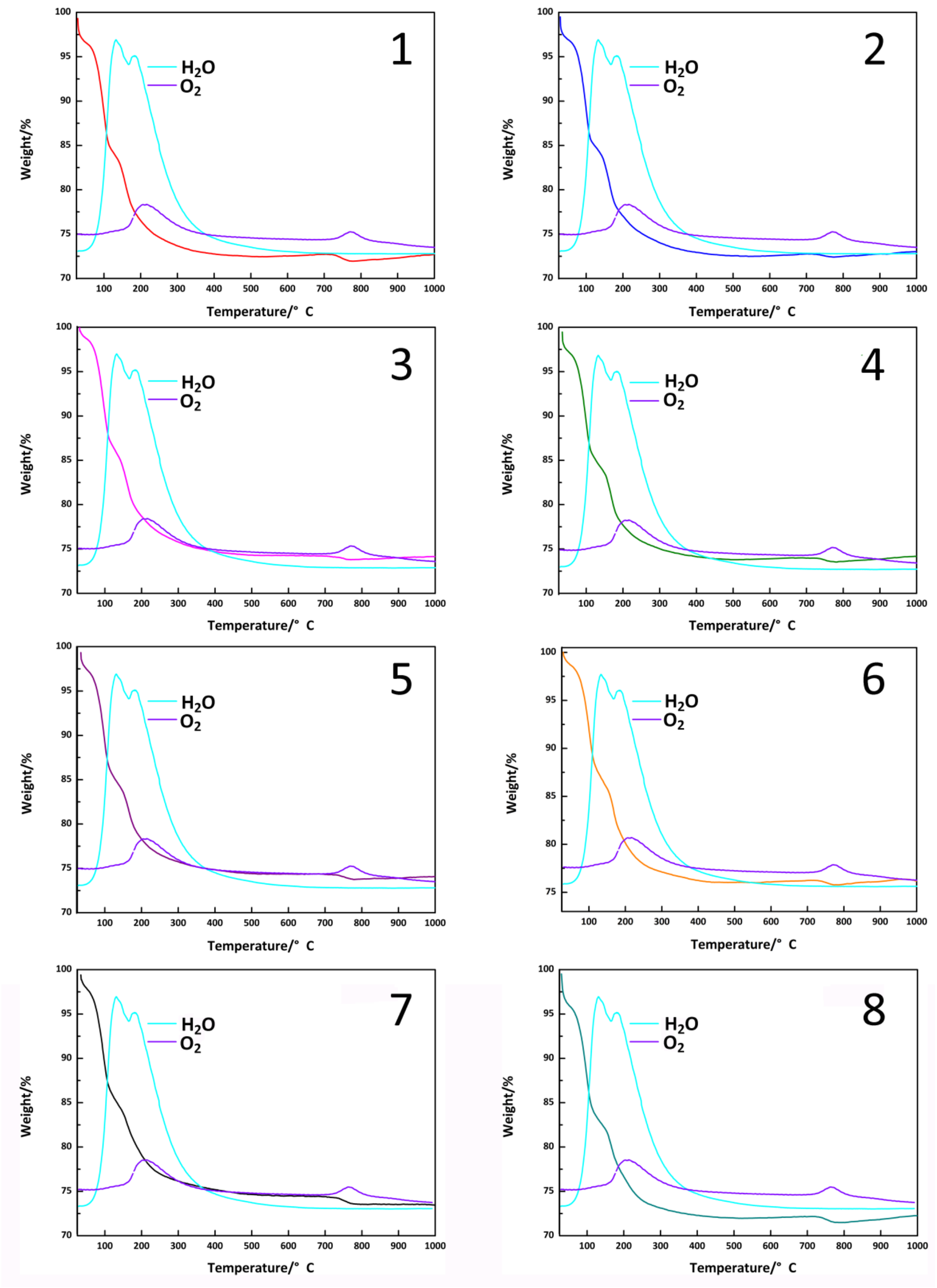

Figure S9. The TG-MS curves of **1−8**.

Thermogravimetric-mass spectrometry (TG-MS) analyses of compounds **1−8** have been performed in the range of 25-1000 °C (Figure S9). The TG curve of compounds **1−8** indicates that the weight loss of **1−8** can be regarded as a two-step weightlessness, corresponding to the

release of twelve or nine lattice water molecules and four protons in the form of aqua ligands as well as the cleavage of six peroxide groups. The total weight loss of experimental value is 25.7%, 25.2%, 25.5%, 24.6%, 25.1%, 24.5%, 25.7% and 24.8% compounds **1−8**, respectively. However, the calculated weightlessness is 33.1%, 32.9%, 32.8%, 32.7%, 32.6%, 30.7%, 32.5% and 32.4% for compounds **1−8**, respectively. The differences may be due to the efflorescence nature of these crystals.

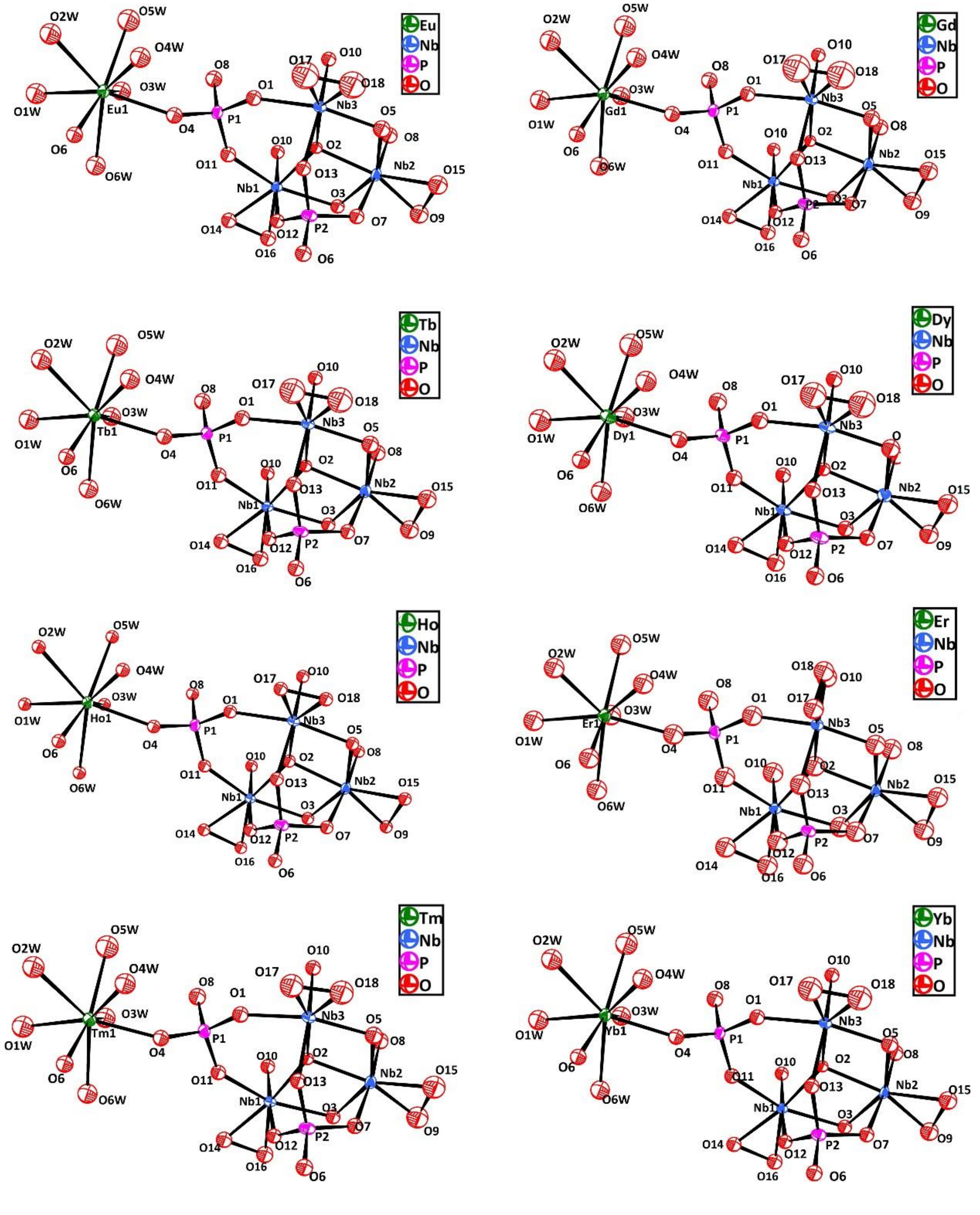

Figure S10. Representations of structural figure with probability ellipsoids of **1−8**.

References

1. Ozeki, T., Yamase, T., Naruke, H. & Sasaki, Y. Synthesis and Structure of Dialuminiohexaeuropiopentakis(hexaniobate): a High-Nuclearity Oxoniobate Complex. *Inorg. Chem.* **33,** 409–410 (1994).
2. Naruke, H. & Yamase, T. $Na_8H_{18}[\{Er_3O(OH)_3(H_2O)_3\}_2Al_2\text{-}(Nb_6O_{19})_5]\cdot 40.5H_2O$. *Acta Crystallogr. C* **52,** 2655–2660 (1996).
3. Naruke, H. & Yamase, T. Structure of dialuminiohexalutetiopentakis(hexaniobate): comparison with europium and erbium analogues. *J. Alloys Compd.* **255**, 183–189 (1997).
4. Yamase, T. & Naruke, H. Luminescence and Energy Transfer Phenomena in Tb^{3+}/Eu^{3+}-Mixed Polyoxometallolanthanoates $K_{15}H_3[Tb_{1.4}Eu_{1.6}(H_2O)_3(SbW_9O_{33})(W_5O_{18})_3]\cdot 25.5H_2O$ and $Na_7H_{19}[Tb_{4.3}Eu_{1.7}O_2(OH)_6(H_2O)_6Al_2(Nb_6O_{19})_5]\cdot 47H_2O$. *J. Phys. Chem. B* **103**, 8850–8857 (1999).
5. Li, C.-C. *et al.* Assembly of Saturated Nb/W Mixed-Addendum Polyoxometalate $[P_2W_{15}Nb_3O_{62}]^{9-}$ and Lanthanide Ions (Ln = Eu, Ce). *Eur. J. Inorg. Chem.* **2012**, 3229–3234 (2012).
6. Li, S.-J. *et al.* Constructing nanosized polyanions with diverse structures by the self-assembly of W/Nb mixed-addendum polyoxometalate and lanthanide ion. *CrystEngComm* **14**, 1397–1404 (2012).
7. Jin, L., Li, X.-X., Qi, Y.-J., Niu, P.-P. & Zheng, S.-T. Giant Hollow Heterometallic Polyoxoniobates with Sodalite-Type Lanthanide-Tungsten-Oxide Cages: Discrete Nanoclusters and Extended Frameworks. *Angew. Chem. Int. Ed.* **55**, 13793–13797 (2016).
